# Do Histologic Features of the Proximal Margin of Resected Specimens Predict Clinical Outcomes in Hirschsprung Disease?

**DOI:** 10.7759/cureus.30809

**Published:** 2022-10-28

**Authors:** Wendy Jo Svetanoff, Sara I Agha, Jason D Fraser, Vivekanand Singh, Atif Ahmed, Rebecca M Rentea

**Affiliations:** 1 Pediatric Surgery, Nationwide Children’s Hospital, Columbus, USA; 2 Pediatric Surgery, Children's Mercy Kansas City, Kansas City, USA; 3 Psychiatry, University of Missouri Kansas City School of Medicine, Kansas City, USA; 4 Pediatric Surgery, University of Missouri Kansas City School of Medicine, Kansas City, USA; 5 Pathology, University of Texas Southwestern Medical Center, Dallas, USA; 6 Pathology, Seattle Children's Hospital, Seattle, USA

**Keywords:** nerve thickness, hirschsprung-associated enterocolitis, clinical pathology, hirschsprung disease, neonatal surgery pediatric gi surgery

## Abstract

Objective

Patients with Hirschsprung disease (HSCR) can experience obstructive symptoms despite adequate resection. We sought to determine if submucosal nerve thickness or length of ganglionated bowel in the resected specimen correlated with functional outcomes.

Methods

A retrospective study of patients who underwent surgery between 2015-2019 was performed. The resected specimen was scanned to measure areas of the thickest submucosal nerves and the length of the ganglionated segment. Functional outcomes were collected via chart review.

Results

Thirty patients were included. The median age at pull-through was 4.5 months (interquartile range {IQR} 0.5 - 6.7 months); 70% were male, and 57% had a Swenson pull-through. The median size of the thickest nerves was 28 micrometers (IQR 24, 32). Three specimens had a nerve thickness of >40 micrometers. The median length of the resected ganglionated segment was 4.4 cm (IQR 2.2, 7.2).

Out of the total, 53% of patients experienced post-operative enterocolitis; 13% required further surgery. At a median of 25.3 months (IQR 17.6, 42.2 months) from pull-through, 33% did not require any bowel regimen therapy. Utilizing logistic regression, neither submucosal nerve thickness nor length of the resected ganglionic segment correlated with outcomes.

Conclusion

While continued bowel management therapy was common, no correlation was found between histologic findings and functional outcomes.

## Introduction

Hirschsprung disease (HSCR) is a congenital aganglionosis of the rectum and colon that extends for a variable distance proximally from the internal anal sphincter (IAS). It is associated with the absence of ganglion cells in the submucosal and myenteric plexus, the absence of mucosal calretinin staining, and an increase in acetylcholinesterase nerve fiber activity [1-4]. The high levels of excitatory neurotransmitters, such as acetylcholine, lead to a spastic contraction of the diseased bowel, causing a functional obstruction [5]. To fully relieve the obstruction, all aganglionic and neurally-abnormal bowel should be resected, pulling through the normally ganglionated colon to just above the dentate line.

The transition zone (TZ) is a segment of the bowel between which ganglion cells first appear and where nerve cells of normal size and diameter occur [2]. Ganglion cells are present in this area, but the neuroanatomy remains abnormal, leading to poor function if used as part of the pull-through procedure. Histological features of the TZ include subcircumferential aganglionosis, myenteric hypoganglionosis, and abnormal extrinsic hypertrophic innervation [6-8]. Criteria for hypertrophic extrinsic nerves include 2+ nerves greater than 40μm in the histological specimen [7]. The subcircumferential aganglionosis is thought to occur for only 3cm proximal to the aganglionic segment leading to recommendations of resecting 5cm beyond the most distal ganglionic biopsy site when performing a pull-through procedure [7-9]. However, the length of the TZ, including the area of partial circumferential aganglionosis, can be variable based on the length of the aganglionic bowel and has been documented to be as high as 30cm in those with long-segment disease [2, 9-11].

It is well documented that patients who have TZ histopathology as part of the pulled-through bowel or have an aganglionic pull-through, have a higher risk of postoperative complications, including constipation, impaction, and enterocolitis [12,13]. Conversely, it is also seen that, despite undergoing complete resection of the aganglionic and TZ colon, patients still suffer from severe constipation or episodes of enterocolitis. Recent research has identified that the number of ganglion cells increases from the distal portion of the TZ to the more proximal portion, but this pattern is not uniform [2]. Likewise, the cholinergic innervation pattern is much less definitive in the more proximal portion compared to the distal TZ, creating difficulties in precisely defining the proximal boundary of the TZ [1,6]. As it is known that complete resection of the TZ leads to improved post-operative outcomes, such as a decreased risk of developing obstructive symptoms or Hirschsprung-associated enterocolitis (HAEC), the question remains: how much normally ganglionated bowel should be resected at the time of the pull-through procedure? We aim to answer this question by determining if submucosal nerve thickness or length of ganglionated bowel in the resected specimen of patients who underwent pull-through for Hirschsprung disease correlated with post-operative functional outcomes, including the development of HAEC, need for redo surgical procedures, need for a bowel management regimen, and the ability to achieve voluntary and/or social continence. This article was previously presented as a meeting abstract at the 2021 EUPSA Annual Congress on September 3, 2021.

## Materials and methods

After institutional review board approval with waiver of informed consent due to the nature of the study, a retrospective review of patients who underwent pull-through (PT) for HSCR at our institution between 2015-2019 was performed. Patients who had their initial pull-through performed at our institution and had pathology specimens of the resected colonic segment available were included. The review occurred between 2020-2021 and consisted of a re-analysis of the resected specimen obtained at the time of the original PT. A pediatric pathologist scanned the resected specimens to measure the length of the ganglionated segment and the average thickness of the submucosal nerves at the proximal margin. The length of the ganglionated segment was measured from the proximal margin to the end of the transitional zone, where normal-appearing ganglion cells and nerves were identified. Nerve thickness was evaluated at cross-sections of the proximal margin, submitted separately or as part of the PT segment, and was measured with Infinity Analyze camera software (Lumenera, Ontario, Canada) attached to a microscope. The complete cross-section of the proximal margin was assessed, and the dimensions of the thickest nerves were measured. A line segment was drawn across the widest diameter or width of the nerve, and the line length was measured in micrometers. Average dimensions were calculated when more than one nerve is found. Demographic data, intra-operative characteristics, and post-operative outcomes were collected via chart review. Post-operative outcomes included the development of Hirschsprung-associated enterocolitis (HAEC), the need for bowel regimen medications at any point after PT, redo surgical interventions, current bowel therapy regimen at last follow-up, and occurrence of any soiling accidents.

All data were analyzed using STATA® (StataCorp, College Station, TX). Categorical data are expressed as percentages and continuous data are expressed as medians with inter-quartile ranges (IQR). Logistic regression was performed to identify if the size of the submucosal nerves or the length of the ganglionated bowel in the resected specimen correlated with the development of enterocolitis, need for further surgery, or obtaining continence of stool. A p-value of < 0.05 was considered statistically significant.

## Results

Thirty patients were included in the analysis. The median age at pull-through was 4.5 months (IQR 0.5 - 6.7 months); 70% were male, and 73% were Caucasian. Seventeen patients (57%) had a Swenson pull-through, while 13 (43%) underwent a Soave procedure; none of the patients had a Duhamel pull-through. The transition zone was located in the rectosigmoid in 18 patients (Table 1). On pathological examination, the median size of the thickest nerves was 28 micrometers (IQR 24, 32). Three specimens had an average nerve thickness of >40 micrometers. The median length of the resected ganglionated segment was 4.4 cm (IQR 2.2, 7.2).

**Table 1:** Demographics of the patients included in our analysis (IQR = inter-quartile range)

	Post Pull-through Patients (n=30)
Gender	
-Male	21 (70%)
-Female	9 (30%)
Race/ Ethnicity	
-Caucasian	22 (73%)
-African American	5 (17%)
-Hispanic	1 (0.6%)
-Asian	1 (0.6%)
-Other	1 (0.6%)
-Multi-racial	0 (0%)
Median Age at Pull-Through	4.5 months (0.5, 6.7)
Type of Pull-Through Procedure	
-Swenson	17 (57%)
-Soave	13 (43%)
Transition Zone	
-Rectosigmoid	18 (60%)
-Descending Colon	7 (23%)
-Transverse Colon	2 (7%)
-Ascending Colon/ TCA	3 (10%)
Duration of Follow-Up	25.3 months (17.6, 42.2)

**Figure 1:**
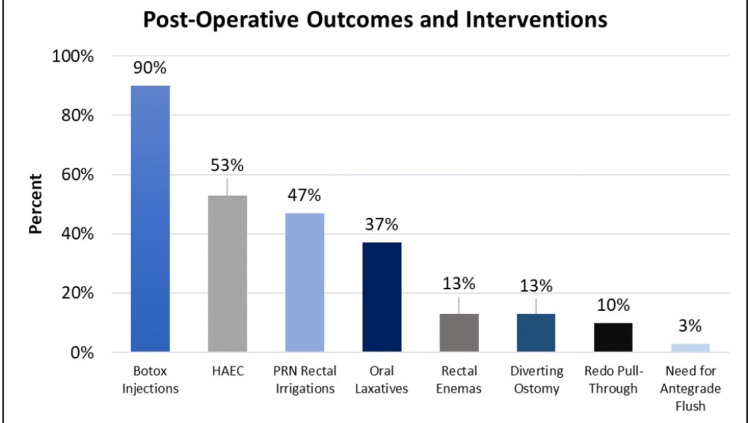
Outcome measures in patients who underwent Swenson or Soave pull-through for Hirschsprung disease PRN = as needed

Sixteen patients (53%) experienced post-operative HAEC, which was defined as admission to the hospital with the performance of rectal irrigations and intravenous antibiotic administration for obstructive symptomatology with or without lethargy, fevers, or poor oral intake; 5 (17%) required further surgery. One underwent antegrade enema access, while four had a diverting ostomy with/without a redo pull-through procedure. Three of the four patients who required a redo operation had developed HAEC after their initial pull-through procedure. The need for a bowel management plan was evident, as 11 patients (37%) required laxatives, and 14 (47%) performed rectal irrigation at home at some time point following pull-through. In line with our recent practice change of performing botulinum toxin (BT) injections to the internal anal sphincter to relieve obstructive symptoms, all but three patients had BT injections performed during their long-term post-operative follow-up [14]. A comparison of outcome measures can be found in Figure 1. At a median follow-up of 25.3 months (IQR 17.6, 42.2 months) from primary pull-through, 10 patients (33%) were not requiring any therapy, 30% still utilized laxatives, and 33% still required intermittent irrigations. Of the 11 patients who were >4 years of age at the latest follow-up, eight (73%) were toilet-trained, and one (3%) was clean of stool with the help of antegrade flushes; the success of toilet training was unknown in two (7%) patients.

Logistic regression was utilized to assess the role that the submucosal nerve thickness and length of ganglionated segment resected played in these postoperative outcomes. Neither submucosal nerve thickness nor length of the resected ganglionic segment correlated with enterocolitis episodes, need for further surgery, or obtaining stool continence (Table 2).

**Table 2:** Logistic Regression analysis looking at post-operative outcomes Logistic regression was performed looking at three post-operative outcomes: incidence of post-operative Hirschsprung-associated enterocolitis, need for redo pull-through, and success in obtaining social continence. The transition zone indicates the area of the colon where the transition zone was located.

Variable	Odds Ratio	95% Confidence Interval	P-value
Post-operative Enterocolitis			
Gender	3.0	0.48 – 18.74	0.24
Race	1.0	0.36 – 2.78	0.99
Transition Zone	0.42	0.16 – 1.12	0.08
Average Nerve Thickness	0.90	0.78 – 1.05	0.19
Length of Ganglionated Segment	0.78	0.55 – 1.10	0.09
Need for Redo Pull-Through			
Gender	1.0	-	-
Race	0.81	0.13 – 5.02	0.82
Transition Zone	0.97	0.30 – 3.14	0.96
Average Nerve Thickness	1.02	0.84 – 1.24	0.84
Length of Ganglionated Segment	0.98	0.87 – 1.10	0.78
Successful Social Continence			
Gender	0.61	0.94 – 3.97	0.61
Race	0.83	0.37 – 1.83	0.64
Transition Zone	1.22	0.45 – 3.29	0.70
Average Nerve Thickness	1.01	0.89 – 1.15	0.82
Length of Ganglionated Segment	1.01	0.93 – 1.09	0.79

## Discussion

In our review of resected specimens of HSCR, we did not find any correlation between the length of the resected ganglionated segment or the size of nerve fibers and postoperative complications, including HAEC, need for redo surgery, or fecal continence. Our study produced similar results to a 2008 study that examined the length of ganglionic and aganglionic bowel with resected colonic specimens in patients with HSCR [15]. In this study, no difference was found in enterocolitis rates among patients who had either >5cm or <5cm of ganglionated bowel resected. There also was no difference in the mean thickness of submucosal nerves between the two groups [15].

In a study performed by Saad et al, the histopathology of 35 patients was examined [4]. Patients were grouped into those who had <3 episodes and those who had ≥3 episodes of HAEC episodes per year. Those who had frequent attacks had significantly more common disarray of nerve bundles and a thicker nerve bundle diameter at the proximal margin of the resected segment [4]. Increased nerve bundle thickness, unmyelinated nerve bundles, and features of acute inflammation at the proximal margin were also associated with poor outcomes. It is thought that these unmyelinated nerve bundles may play a role in decreased colonic motility, potentially leading to constipation and obstructive symptoms [4]. This indicates that the transition zone can be problematic to define; even in our study, three patients were found to have some hypertrophic nerves at the resected margin. However, extra vigilance should be taken in caring for these patients as it is unclear whether this leads to a higher incidence of postoperative complications. In our study, all three patients with a nerve thickness >40µm required Botulinum toxin injection into the internal anal sphincter, and one patient required a redo pull-through. Finally, in a retrospective review published in 2020, the length of the TZ was compared to the need for revisional surgery in 48 patients [16]. No difference was found among patients who had a transition zone >5cm or ≤5cm when focusing on postoperative complications or the need for revisional surgery [16]. While our study did not look specifically at the length of the TZ, we found no difference in the length of the ganglionated segment proximal to the TZ and the development of postoperative complications.

It is well known that patients with a transition zone pull-through (TZPT) have a higher risk of complications, such as HAEC, as the TZ can result in obstructive symptomatology [2,12]. In a systematic review of patients who underwent a redo PT, 35% had abnormal findings on rectal biopsy, including 49/193 who had a TZPT; these patients most commonly presented with constipation and recurrent HAEC [17]. Similar findings were seen in another study, where they looked at 93 patients who required revisional operations [13]. Twenty-five patients had residual aganglionosis or TZPT, and the prominent symptoms of all patients were enterocolitis (56%) and constipation (44%). Thus, all patients who present with recurrent obstructive symptoms should undergo a repeat rectal biopsy to confirm the presence of ganglion cells at the level of anastomosis.

However, even patients who undergo an appropriate PT may still develop obstructive symptoms and HAEC, and we are unable to predict which patients will develop these complications accurately. In a multicenter retrospective review of nearly 300 patients by the Pediatric Colorectal and Pelvic Learning Consortium (PCPLC), patients with a rectosigmoid transition zone had a lower association with postoperative HAEC compared to a more proximal transition zone [18]. Other potentially important variables, such as insurance status and incidence of preoperative HAEC were not found to be associated with postoperative HAEC on multivariate analysis; the authors concluded that further multi-institutional studies were needed to identify other potential risk factors [18]. In our study, we were unable to find any correlation in the length of the resected ganglionic segment or in the size of the nerve fibers with postoperative functional outcomes; however, we presume that there may be other histological factors that we have not examined could be important risk factors.

Studies that have attempted to predict which patients will develop such symptoms have focused on the histopathology of the resected colonic specimens. In one retrospective review that assessed the pattern of cholinergic innervation and the number of ganglion cells in resected specimens, no definitive pattern could be found between the amount of cholinergic innervation and ganglion cells [1]. Another observational study evaluated the extent of the TZ in patients with HSCR; no relationship was found between the pattern of distribution of nerve trunk hypertrophy and the distribution of hypoplastic ganglia [2]. Altered expression of neurotransmitters also has been seen in the bowel of patients with HSCR [5]. In a study by Coyle et al., neuronal nitric oxide synthase was found to be higher in the ganglionated bowel of patients with HSCR and reduced in the aganglionic segment compared to the bowel of patients without HSCR, while choline acetyltransferase was higher in aganglionic bowel and lower in ganglionated bowel compared to controls [5]. The inverse relationship between nitric oxide synthase and choline acetyltransferase expression was identified as a gradient traveling from the aganglionic bowel to the ganglionated bowel within the resected specimens. The overexpression of choline acetyltransferase leads to the spastic contraction of the aganglionic segment, while the neuronal nitric oxide synthase is believed to be responsible for the hypomotile megacolon that may contribute to persistent symptoms in patients after pull-through [5]. Future research in this area should focus on how different expression levels of these neurotransmitters play a role in postoperative outcomes, such as HAEC, severe constipation, and the need for revisional surgery.

Patients with HSCR who require a bowel management program have different considerations compared to those who have an anorectal malformation. Factors related to soiling and incontinence in the HSCR population include both the motility of the colon (hypermotile vs hypomotile) as well as the post-surgical anatomy, specifically if there has been damage to the sphincters and if the patient has sensation in the anal canal [19]. It is believed that delaying the definitive pull-through until the patient is 3-4 months of age will allow for better identification of perineal anatomy and avoidance of sphincter damage; however, a retrospective review of 82 patients with HSCR within the PCPLC found no difference in short vs long-segment disease, preoperative or postoperative HAEC episodes, or in need for bowel management between those who underwent pull-through in the neonatal period compared to those who underwent pull-through in a delayed fashion [20]. The 48.5% rate of enterocolitis in the delayed group is similar to our findings, where 57% experienced HAEC after undergoing a pull-through procedure at a median of 4.5 months of age.

Limitations of this study include its retrospective design and identifying variables that may be important in predicting postoperative complications was limited by available documentation in the electronic medical record. Due to the retrospective nature of this review, objective measures of functional outcomes could not be calculated as aspects of previously validated scoring systems were not routinely documented in the patient's charts during follow-up. Therefore, we used continence as a surrogate for functional outcomes as the ultimate goal of all children with Hirschsprung disease is to remain either voluntarily or socially continent. Previous studies have shown that a criterion of >40 μm for hypertrophic nerves may not be fully accurate after the patient reaches one year of age [9,21]. We also did not look at expression levels of neurotransmitters within the proximal portion of the specimen; this may be an area of future research to determine if differences in expression correlate with an increased risk of postoperative obstructive symptoms.

## Conclusions

Patients with Hirschsprung disease can experience obstructive symptoms despite adequate resection. These symptoms can lead to the development of enterocolitis, affect the patient’s ability to achieve fecal continence, and may result in further procedures in order to allow for better stooling patterns. What is unclear is whether the length of the ganglionated bowel in the resected specimen or the thickness of the submucosal nerves correlates with functional outcomes, specifically the presence of obstructive symptomatology and the ability to achieve continence.

In our retrospective review, no correlation was found between histologic findings (submucosal nerve thickness or length of ganglionated bowel in the resected specimen) and postoperative functional outcomes following pull-through. The need for continued bowel management therapy was common; however, all patients greater than age four years in whom toilet training status was known were socially continent.
